# Rabies Virus Hijacks and Accelerates the p75NTR Retrograde Axonal Transport Machinery

**DOI:** 10.1371/journal.ppat.1004348

**Published:** 2014-08-28

**Authors:** Shani Gluska, Eitan Erez Zahavi, Michael Chein, Tal Gradus, Anja Bauer, Stefan Finke, Eran Perlson

**Affiliations:** 1 Department of Physiology and Pharmacology, Sackler Faculty of Medicine, and the Sagol School of Neuroscience, Tel Aviv University, Tel Aviv, Israel; 2 Friedrich-Loeffler-Institut, Federal Research Institute for Animal Health, Institute of Molecular Virology and Cell Biology, Greifswald – Insel Riems, Germany; Thomas Jefferson University, United States of America

## Abstract

Rabies virus (RABV) is a neurotropic virus that depends on long distance axonal transport in order to reach the central nervous system (CNS). The strategy RABV uses to hijack the cellular transport machinery is still not clear. It is thought that RABV interacts with membrane receptors in order to internalize and exploit the endosomal trafficking pathway, yet this has never been demonstrated directly. The p75 Nerve Growth Factor (NGF) receptor (p75NTR) binds RABV Glycoprotein (RABV-G) with high affinity. However, as p75NTR is not essential for RABV infection, the specific role of this interaction remains in question. Here we used live cell imaging to track RABV entry at nerve terminals and studied its retrograde transport along the axon with and without the p75NTR receptor. First, we found that NGF, an endogenous p75NTR ligand, and RABV, are localized in corresponding domains along nerve tips. RABV and NGF were internalized at similar time frames, suggesting comparable entry machineries. Next, we demonstrated that RABV could internalize together with p75NTR. Characterizing RABV retrograde movement along the axon, we showed the virus is transported in acidic compartments, mostly with p75NTR. Interestingly, RABV is transported faster than NGF, suggesting that RABV not only hijacks the transport machinery but can also manipulate it. Co-transport of RABV and NGF identified two modes of transport, slow and fast, that may represent a differential control of the trafficking machinery by RABV. Finally, we determined that p75NTR-dependent transport of RABV is faster and more directed than p75NTR-independent RABV transport. This fast route to the neuronal cell body is characterized by both an increase in instantaneous velocities and fewer, shorter stops en route. Hence, RABV may employ p75NTR-dependent transport as a fast mechanism to facilitate movement to the CNS.

## Author Summary

Rabies virus (RABV) is a neurotropic virus that depends on long distance axonal transport in order to reach the central nervous system (CNS). The strategy RABV uses to hijack the cellular transport machinery is unknown. Here we use live cell imaging to track RABV entry at nerve terminals and study its retrograde transport along the axon. First, we demonstrate that RABV interacts with the p75 neurotrophin receptor (p75NTR) at peripheral neuron tips to enter the axon. Then, characterizing RABV retrograde transport along the axon, we showed that the virus moves in acidic compartments, mostly with p75NTR. Interestingly, RABV is transported faster than NGF, an endogenous p75NTR ligand. Finally, we determine that p75NTR-dependent transport of RABV is faster and more directed than p75NTR-independent RABV transport. Hence, RABV not only exploits the neurotrophin transport machinery, but also has a positive influence on transport kinetics, thus facilitating its own arrival at the CNS.

## Introduction

Rabies virus (RABV) is a neurotropic negative-strand RNA virus of the *Lyssavirus genus*, belonging to the *Rhabdoviridae* family. It is transmitted mostly via bites of diseased animals and causes a fatal infection of the nervous system in both animals and humans. A key step in RABV pathogenesis is rapid transfer to the Central Nervous System (CNS) through the Peripheral Nervous System (PNS) [1]. Due to its extraordinary properties in directed axonal transport and trans-synaptic spread, RABV has also been used as a neuro-tracing agent to map neuronal circuitry [2]–[5]. Thus, understanding the mechanism of RABV transport is of high significance for both basic and applicative fields.

RABV enters the peripheral nervous system and undergoes long-distance transport arriving at the cell soma and subsequently the CNS [6]. As peripheral neurons are highly polarized cells with long axons, active intracellular transport is vital to the maintenance of neuronal function and survival [7], [8]. Axonal transport is the cellular process of trafficking proteins, organelles, vesicles, RNA and other cellular factors to and from the neuronal cell body. The molecular motor kinesin drives transport from the cell body anterogradely, supplying proteins, lipids and other essential materials to the cell periphery. Dynein/dynactin complexes drive retrograde transport, moving damaged proteins for degradation and critical signaling molecules such as neurotrophins to the cell body [9], [10]. Although RABV phosphoprotein P, a component of the viral nucleocapsid of infecting virions, was shown to directly interact with a light chain of the dynein motor complex [11], [12], axonal RABV transport and CNS infection are independent of that interaction [13] and long distance transport of complete enveloped virions within internalized endosomes is more likely [14]. However, the cellular and molecular mechanisms involved in RABV's infection and retrograde trafficking are yet to be understood.

Entry of RABV into the cell requires binding of the viral glycoprotein (G) and fusion of the virus envelope with the host cell membrane [15]. Following receptor binding and fusion, RABV may enter the host cell through the endosomal transport pathway. In neurons, infected cells may mistake RABV particles for cargo and thus recruit trafficking components, allowing viral particles to undergo long-range axonal transport to the neuronal cell body, as was found in the case of adenovirus and the CAR receptor [16], [17]. Direct evidence for this notion is still lacking for RABV, as well as the identity and role of the molecular determinants of the axonal transport machinery RABV utilizes.

Both the Neuronal Cell Adhesion Molecule (NCAM) and the p75 neurotrophin receptor (p75NTR) have been identified as RABV glycoprotein G binding receptors [18], [19]. Other membrane-associated components have also been implicated in RABV binding [20]. By binding one of its receptors, RABV could enter the cell and activate downstream signaling which would allow it to hijack and manipulate axonal transport machineries. Although p75NTR is known to be involved in the retrograde transport of neurotrophic factors, little is known regarding its direct contribution to viral transport. It was recently shown, however, that lentiviral vectors pseudotyped with RABV-G are retrogradely transported in motor neurons and co-localize with both p75NTR and NCAM [21]. The p75NTR contains four cysteine-rich domains (CRD) in the N-terminal ectodomain and a type II death domain in its cytoplasmic C-terminal segment. Rabies virus glycoprotein specifically interacts with high affinity with the first Cysteine-Rich Domains (CRDI) of p75NTR [22]. Neurotrophins, on the other hand, bind to the second and third p75NTR cysteine-rich domains (CRDII&III) [23]. Hence, RABV and neurotrophins do not compete for each other's binding site. However, it was previously reported that treatment of cells with NGF and Neurotrophin-3, ligands of p75NTR, modulates RABV infection of DRG-originated neurons [24]. Remarkably, although p75NTR binds RABV with high affinity, it is not essential for its infection [25], further raising questions regarding the specific role of this interaction.

Here we study the strategies used by RABV to exploit axonal transport mechanisms during CNS invasion. We tracked RABV entry at nerve terminals and studied its retrograde transport along the axon in comparison to the transport of NGF. We show that RABV and NGF are internalized in similar time frames at similar domains along nerve tips and that RABV enters the cell along with p75NTR, suggesting common entry machineries. Then, by tracking the transport of GFP labeled Rabies virions along the axon, we showed it moving in acidic compartments, mostly with neurotrophic factor receptors, yet faster than NGF. Finally, we determined that p75NTR-dependent transport of RABV is faster and more directed than p75NTR-independent transport. Our model suggests that RABV may enter the cell by receptor-mediated endocytosis following its binding to p75NTR, after which it enhances the efficiency of the retrograde co-transport of RABV – p75NTR complexes. The interaction with p75NTR modulates the cellular transport machinery and serves as a mechanism to facilitate movement of RABV to the CNS.

## Results

### RABV and NGF undergo retrograde axonal transport in sensory axons

**Figure 1:**
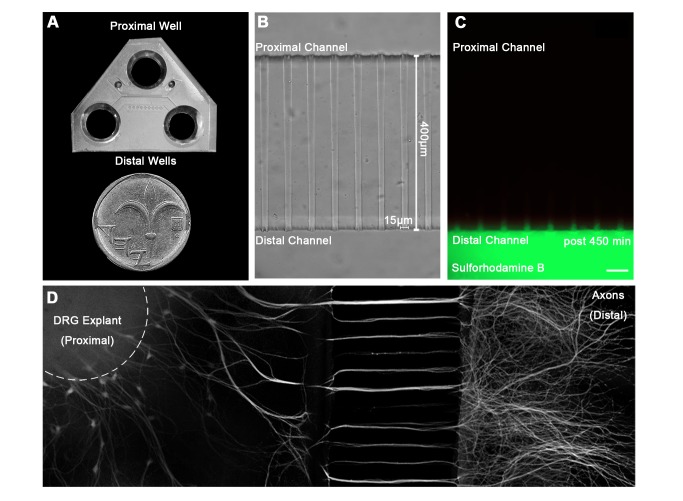
A microfluidic system for tracking retrograde transport in sensory axons. (**A**) A Polydimethylsiloxane (PDMS) microfluidic chamber used for explant culture. (**B,C**) 40 µl interval towards the proximal compartment (top) prevents fluorescent dye from diffusing to the proximal compartment, allowing several hours of compartmental separation. Bright field (**B**) and fluorescent images (**C**) taken 7.5 hours after addition of Sulforhodamine B fluorescent dye to the distal compartment (bottom). (**D**) DRG explants are healthy and extend axons through micro-grooves to distal compartment after 2–3 days in vitro. One microgroove typically contains 2–5 axons. Mosaic of 10× images of Calcein-stained DRG explant taken after 5 DIV. Scale bar = 50 µm.

**Figure 2:**
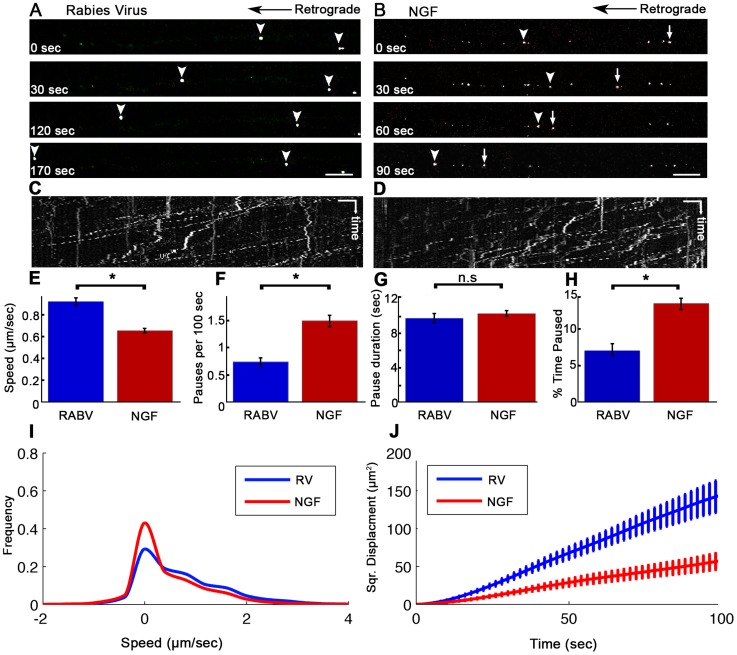
Rabies virus retrograde transport in DRG is faster and more directed than that of NGF. Retrograde transport of (**A**) EGFP-RABV and (**B**) Quantum-dot conjugated NGF in DRG explants, roughly 2 hours after addition to distal axon compartment. Arrows and arrowheads pointing at transported particles. Scale bars = 10 µm. (**C,D**) Kymograph for EGFP-RABV and NGF respectively. Horizontal scale bars = 5 µm, vertical scale bars = 40 seconds. (**E–J**) Characterization of manually tracked directed particles of RABV (n = 209) and NGF (n = 149). (**E**) RABV is transported faster than NGF, as seen by average speed. (**F**) RABV is more directed and pauses less than NGF (**G**) Average pause duration was not significantly different. (**H**) RABV spent a smaller fraction of its run paused (**I**) Instantaneous velocities of RABV particles (n = 9885 events) are higher than those of NGF particles (n = 8973 events). Positive and negative values represent retrograde and anterograde velocities, respectively. (**J**) RABV particles travel larger net distances than NGF's, as seen by mean squared displacements. Data was pulled from two separate experiments. Error bars represent SEM, *p<0.0001.

In order to study the mechanism of RABV long distance transport, we used an optimized compartmentalized microfluidic culture chamber. In this system, murine E12.5–13.5 DRG explants were plated in one side of the chamber, referred to here as the proximal channel (Fig. 1A,B). Explants are encouraged to extend axons to the distal axon channel through microgrooves by introduction of a gradient of NGF known to promote DRG axonal growth (Fig. 1D). By maintaining a difference in media volume we induce directional flow across grooves or channels. Thus, fluorescent dyes like Sulforhodamine B, introduced into the distal channel that contains less medium, are prevented from reaching the grooves or proximal channel where cell bodies are located (Fig. 1C). Hence, EGFP-RABV added to the axon terminus, binds exclusively to the distal axon, enabling retrograde tracking of the virus along axons in the groove. Following serum and trophic factor starvation, EGFP-RABV virions were introduced into the distal channel and 1–2 hours later were observed to move retrogradely towards the cell body, as seen by time lapse imaging (Fig. 2A,C and Movie S1). X-Y coordinates of particles moving over time were manually registered and compiled into tracks. 87.29% of RABV particles (n = 244), were visible over at least 10 consecutive frames and had an average instantaneous velocity >0.1 µm/sec. These were considered directed particles and characterized further (Fig. 2E–J).

Since RABV-G is known to bind the p75NTR neurotrophin receptor, we asked whether RABV exploits NGF's endogenous transport machinery in order to facilitate its own transport to the cell body, and eventually to the CNS. To address this question, we applied Quantum-Dot conjugated NGF to axon tips in the distal side of the chamber after starvation, and tracked its retrograde transport along grooves (Fig. 2B,D and Movie S2). Detailed transport analysis of RABV and NGF puncta along axons, demonstrates that RABV moves significantly faster. The average speed of RABV was roughly 40% higher than that of NGF (0.93±0.03 versus 0.66±0.02 µm/sec, respectively) (Fig. 2E). While both RABV and NGF particles presented a “stop and go” motion (Fig. 2C,D) RABV particles demonstrated a more processive movement with fewer stops (Fig. 2F). Though spot duration did not differ significantly (Fig. 2F). RABV particles spent a larger percentage of their traffic time in direct movement (Fig. 2H). Moreover, RABV's instantaneous velocity distribution profile was shifted towards the higher velocities (Fig. 2I), averaging at 0.785±0.008 µm/sec, while the average for NGF was 0.546±0.007 µm/sec (n = 7318 and 5452, respectively). Hence, RABV moves faster and travels longer distances (Fig. 2J). These findings suggest that RABV not only exploits the axonal mechanism for neurotrophin transport, but might also increase transport efficiency.

Explants grown in microfluidic chambers tend to vary in the axonal meshwork formed at the distal channel, and consequently in the number of viral particles found in each groove. We therefore checked whether the number of tracked particles per groove had an effect on measured values. We found no correlation between number of EGFP-RABV puncta tracked per axon and that of percentage of directed puncta, their speed, displacement or run length (Fig. S1).

### RABV and NGF are internalized at the axon tip with comparable kinetics

In order to illustrate the differences leading to faster transport of RABV compared to NGF, we proceeded to inquire whether internalization of these ligands occurs over similar time frames. To this end, we performed a series of live imaging experiments using TIRF microscopy, and tracked fluorescent RABV or NGF particles at the axonal growth cone. Distinct features of the TIRF evanescent wave allow us to limit our view to the basal surface, an ideal set up for viewing internalization processes occurring at axon tips.

**Figure 3:**
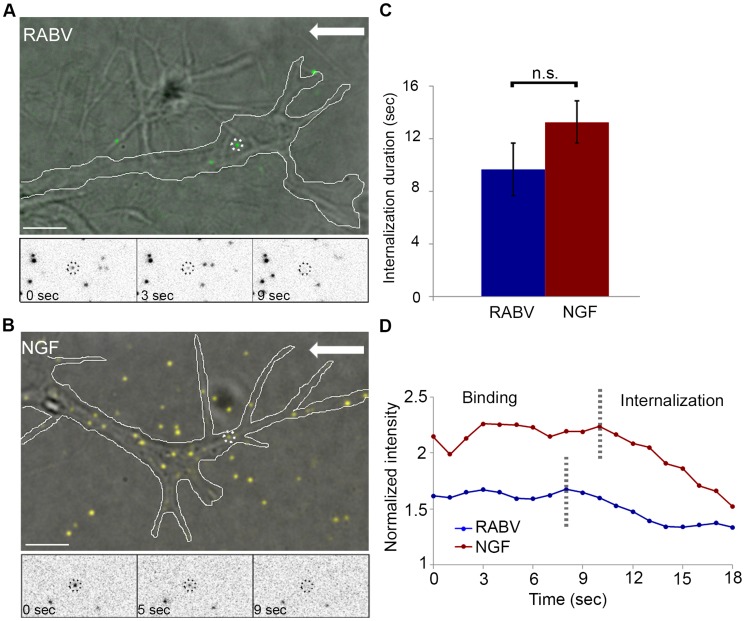
RABV and NGF present similar internalization kinetics at the axon tip. Live TIRF microscopy was used to track RABV and NGF internalization in DRG neuron tips. (**A**) EGFP-RABV (dashed circles) is detected on the surface of a neuron tip (white line). Lower panels present a particle (circled in black) disappearing gradually into the cell over a course of ∼9 seconds. (**B**) Qdot labeled NGF undergoes gradual internalization at the tip of the DRG neuron. (**C**) Average internalization time of RABV and NGF (n = 6 and 8, respectively), from onset of gradual signal reduction to its disappearance, do not differ significantly [p = 0.148]. (**D**) Representative time course of individual RABV (blue) and NGF (red) particles intensity profile, from detection on the cell surface to their internalization. Arrows represent direction of the neuron soma. Scale bars = 5 µm.

Fluorescently labeled RABV or NGF was applied to DRG explant cultures and their dynamics at the axon tips were tracked (Fig. 3). Both RABV and NGF particles demonstrated similar internalization profiles. They arrived at the axon extremity, anchored to the cell membrane and then travelled for a few seconds before eventually internalizing into the cell. Internalization was manifested as a gradual decrease in particle intensity until complete disappearance (see methods for more details), (Fig. 3A,B and Movies S3,S4). Quantification of internalization durations revealed similar kinetics for RABV and NGF (Fig. 3C,D) (9.66±1.97 seconds and 13.25±1.58 seconds, respectively, p = 0.148).

### RABV binds the p75 neurotrophin receptor at the DRG axon tip prior to co-entry into the cell

**Figure 4:**
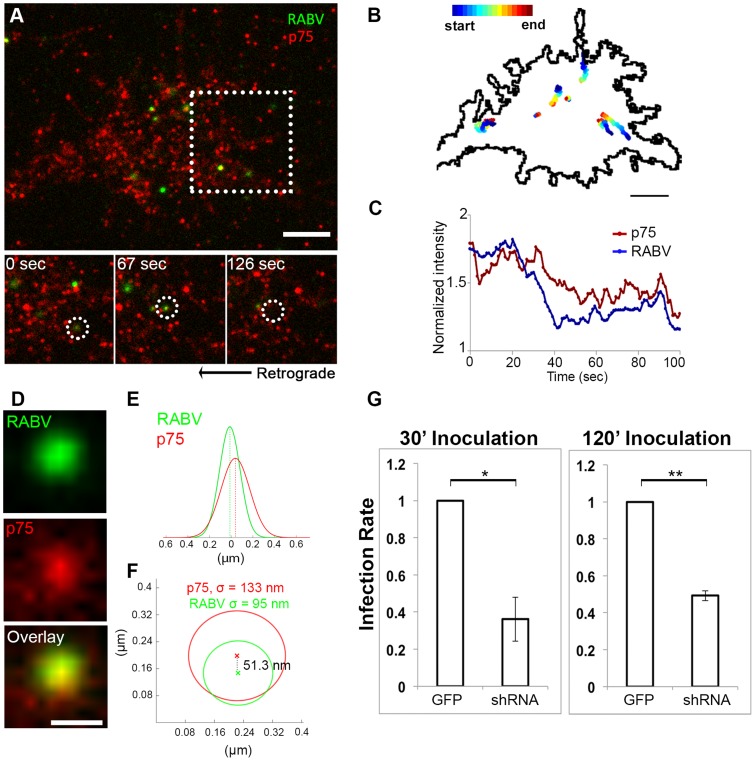
RABV binds and internalizes with p75NTR in DRG neuron tips. Co-localization of EGFP-RABV with p75NTR is shown by live TIRF imaging and sub-pixel localization algorithms. (**A**) RABV-p75 particles shift from the periphery to the center of the growth cone, where they are internalized into the cell. Lower panels zoom in on dashed square, showing co-localized puncta (left) shifting towards the center of the growth cone (middle) until finally internalized (right). (**B**) Presentation of six separate events of RABV and p75NTR binding and internalization on the surface of the growth cone shown in (A). Colored trajectories denote displacement from point of detection to point of disappearance. (**C**) RABV and p75NTR are internalized together, illustrated by corresponding plots of puncta intensity over time (normalized to background), calculated for co-localized particles shown in lower panels of (A). Scale bars = 5 µm. (**D**) Zoom-in on colocalized RABV and p75 spot, taken from panel (A), scale bar = 1 µm. (**E**) Overlay of 1D-Gaussian fits of p75 and RABV intensity profiles at the x-axis of the image in panel (D). (**F**) Representative overlay of radial symmetry fits of the x-y intensity profiles of p75 and RABV spots. σ is the standard deviation of each fitting function; distance between the two spot centers is 51.3 nm. (**G**) Knockdown of p75NTR decreases rabies virus infection for shorts time incubation. DRGs embryonic cells infected with lentiviral vectors (LV) containing 4 different EGFP-tagged shRNA's against p75NTR or LV-EGFP, were transfected with RABV for 30 or 120 minutes. Low levels of infected neurons were found in shRNA-p75-EGFP cells Average RABV infection rates were normalized to LV-EGFP controls (n = 4 experiments, error bars = SEM, *p<0.005, **p<0.0005).

RABV-G was shown to bind the p75NTR with high affinity [22], yet there is no direct evidence demonstrating that p75NTR can act as a receptor to mediate RABV internalization. We therefore conducted a second series of live TIRF imaging assays where EGFP-RABV was applied to DRG explant cultures along with a fluorescent antibody against the extracellular domain of the p75 receptor. Dual color TIRF live imaging revealed that RABV and p75NTR were dynamically co-localized at the cell membrane, and were internalized together at the axon tips (Fig. 4A,C and Movie S5). Interestingly, tracking dual-color particles indicated that these followed a directed path towards the center of the axonal growth cone prior to their internalization (Fig. 4B). Thus, RABV binds and is internalized at the axon tip together with p75NTR in a manner similar to that of NGF. In order to further validate the intimate proximity of p75 and RABV on the cell surface, we used single particle localization algorithms, to determine the relative position of co-localized RABV and p75 spots from live TIRF images at subpixel resolution using Gaussian and radial symmetry fitting (Fig. 4D–F). Distances between p75 and RABV were measured according to the center positions of the radial symmetry fits, using Parthasarathy's Radial Center algorithm [26], and averaged on 85.5±20.82 nm (n = 7). This measurement may reflect the actual distance between the rabies virion, roughly 100×200 nm in size, and the p75NTR it binds. Other factors, such as the size of the labeling antibody and imperfect optical alignment may have also contributed to the measured distance. Co-localization was further confirmed by stimulated emission depletion (STED) microscopy, on DRG explants were treated with either a combination of RABV-EGFP and anti-p75-550, or RABV-mCherry and anti-p75-488 (Fig. S2).

Our findings redirect attention to the question of p75NTR's role in RABV infection, in light of previous studies which have shown that p75NTR is not essential for RABV infection but afects clinical manifestation [27]. To address this question, we measured RABV infection rates in p75NTR-knocked down DRG cultures. Mixed infection with 4 shRNA constructs against p75NTR decreases p75NTR levels in DRGs (Fig. S3). Short inoculation times of 30 and 120 minutes were chosen, to study p75NTRs role in promotion of infection. Lower infection rates were observed in cultures infected with shRNA as opposed to GFP infected controls (Fig. 4G), at both time points. Under these conditions, p75NTR expression enhances RABV infection of embryonic sensory neurons. Similar observations were made upon p75NTR knockdown in the NSC34 motor neuron cell line (data not shown).

### RABV and NGF are co-transported in DRG axons

**Figure 5:**
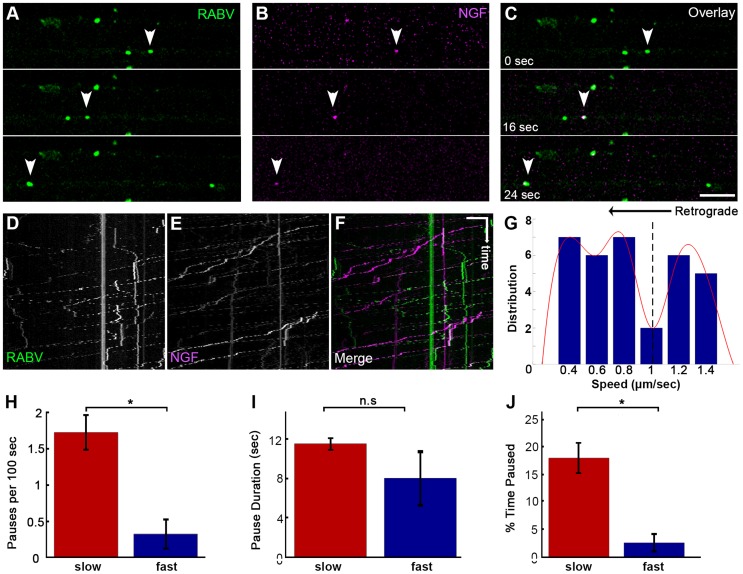
Co-transport of RABV and NGF. (**A–C**) EGFP-RABV and Qdot-NGF were simultaneously added to the distal compartment of a DRG explant at 3DIV. Dual-channel live imaging revealed multiple events of RABV co-transported with NGF, illustrated by corresponding images from either channel and overlay. Arrowheads: co-localized puncta trafficked over time. Scale bar = 10 µm. (**D–F**) Kymographs of EGFP-RABV and Qdot-NGF show mutual transport of RABV and NGF. (**G**) Two populations of RABV-NGF particles were identified by distribution of average track speeds, as seen by fitted curve (red, minima point represented by dashed line, n = 46). (**H–J**) “Slow” (n = 38) and “fast” (n = 8) populations according to the minima (1.01 µm/sec) in (G). “Slow” tracks paused more than “fast” tracks (**H**), and though stop duration was not significantly different (**I**), spent a larger fraction of their travel paused (**J**). Data is pulled from two experiments. Error bars represent SEM, *p<0.02.

As both RABV and NGF traffic retrogradely to the cell body, and use a similar internalization process, we asked whether RABV hijacks the NGF endosomal retrograde transport machinery. To address this issue we performed dual color live imaging of RABV and NGF retrograde axonal transport in microfluidic chambers. Tracking RABV and NGF, we analyzed ∼50 events in which RABV and NGF were retrogradely transported together in the same compartment along the axon (Fig. 5A–F and Movie S6). Examining the distribution of average track speeds we found that the co-transported RABV/NGF particles could be divided into two populations (Fig. 5G). Separate characterization of these two populations shows that faster tracks were less prone to pausing mid-way (0.3±0.2 versus 1.7±0.2 pauses per 100 seconds, respectively). As only 2 pauses were recorded in the fast group, no significant difference was found between either group's stop durations (Fig. 5H,I). Overall, the fast group, thus spent less time paused (Fig. 5J). The presence of two distinct populations of RABV/NGF co-transport may suggest to a switch in “drivers”, where NGF leads the slower group while the faster is led by RABV.

### RABV is retrogradely transported in an acidic compartment in DRG axons

**Figure 6:**
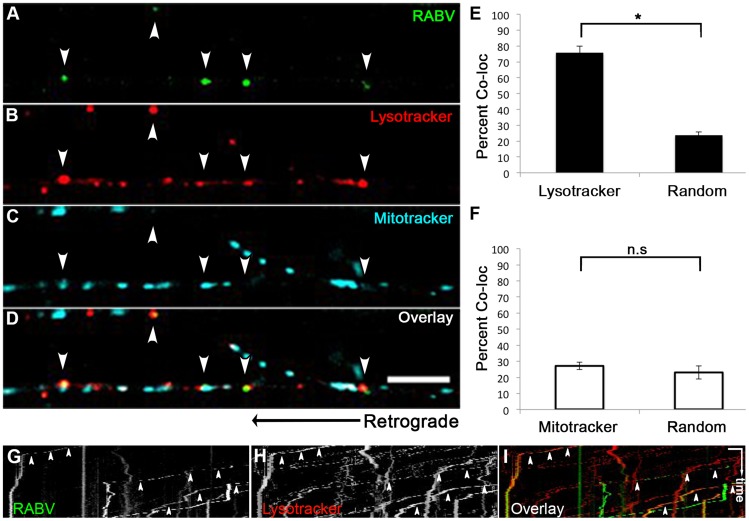
RABV is transported in acidic compartments in sensory axons. (**A–D**), Multi-channel live imaging of EGFP-RABV retrograde transport in DRG axons, along with the fluorescent cellular markers Lysotracker Red and Mitotracker Deep Red. Arrowheads: RABV particles positive for Lysotracker Red. Scale bar = 10 µm. (**E**) 75.9% of RABV puncta are co-localized with Lysotracker Red, thus found in acidic compartments along the axon, as opposed to 23.6% co-localization with random Lysotracker frames from the same time lapse (n = 3 separate experiments). (**F**) RABV does not co-localize with Mitotracker. (**G–I**) Kymographs of EGFP-RABV (**G**) and Lysotracker (**H**) are overlaid in (**I**) show kinetics of RABV (green) and Lysotracker (red) retrograde transport in DRG axons. Arrowheads: acidic transport of RABV. Horizontal scale bar = 5 µm, vertical scale bar = 40 seconds. *p<0.0005. n.s = non significant.

We proceeded to further characterize the transport mechanism of RABV particles; seeking to first determine the cellular compartment in which RABV particles are transported, post infection, within DRG axons. Using microfluidic chambers (Fig. 1), we infected axons in the distal compartments, while simultaneously treating cells with fluorescent markers of cellular compartments (Fig. 6). In order to check that RABV is transported in acidic compartments [28], possibly late-endosomes, lysosomes or autophagosomes, and to quantify this co-localization, we treated cells with the PH-indicator dye, Lysotracker Red. In order to observe whether RABV is transported with mitochondria, we treated cells with the Mitotracker Deep Red marker. Using both markers together with EGFP-RABV, we acquired three channel time- lapse image series of RABV transport (Fig. 6A–D). Detection and analysis of co-localized fluorescent spots along the axon determined that most of the RABV particles (75.9±4.09%, n = 3 separate experiments) were located in acidic compartments (Fig. 6E). Examination of merged kymographs of RABV and Lysotracker red amplified the outcome of co-localization analysis, as most transient RABV tracks were matched with a Lysotracker red track (Fig. 6G–I). Thus, RABV particles are located in acidic axonal endosomes that retrogradely move towards the cell body. In contrast, we could not detect any significant co-localization of RABV and mitochondrial marker (Fig. 6F). We therefore conclude that while RABV is mostly transported in an acidic compartment, it is not transported along with mitochondria in DRG axons.

### RABV is transported with neurotrophin receptors

**Figure 7:**
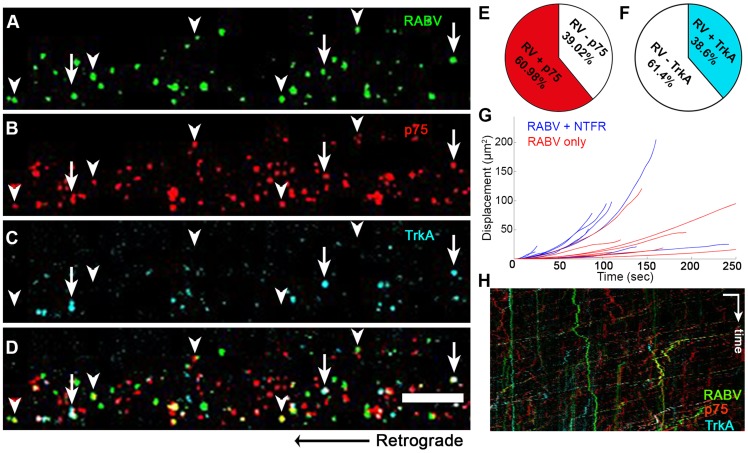
RABV is retrogradely transported with neurotrophin receptors. (**A–D**), Retrograde transport of EGFP-RABV, added to the distal axon compartment of DRG explant previously treated with fluorescent antibodies against p75NTR and TrkA. Arrowheads: RABV puncta positive for p75NTR, arrows: RABV puncta positive for p75NTR and TrkA. Scale bar = 10 µm. (**E,F**) Co-localisation of RABV with p75NTR and TrkA calculated from two and one experiments, respectively. (**G**) Trajectories of RABV trafficked with neurotrophin receptors (NTFR, Blue) or without (Red), illustrating a more processive displacement over time of RABV with NTFR. (**H**) Merged kymographs of RABV (green) p75NTR (red) and TrkA (cyan), drawn for multi-channel time lapse. Vertical scale bar = 5 µm, horizontal scale bar = 40 seconds.

Having determined that RABV is internalized with p75NTR and can undergo transport with NGF, we then tested whether it is also transported with more selective neurotrophin receptors. In order to study this, we infected DRG axons grown in microfluidic chambers with EGFP-RABV, while labeling these cells with fluorescent antibodies against neurotrophin receptors. Specifically, we used fluorescent-tagged antibodies against the general neurotrophin receptor p75NTR and the specific NGF receptor TrkA and acquired three-channel time-lapse movies of RABV and these receptors (Fig. 7A–D). Co-localization analysis determined that the over 60% of RABV particles co-localized with p75NTR, yet less than 40% with TrkA (Fig. 7E,F,H and Movie S7). Interestingly, when co-localized with neurotrophic factor receptors, mainly p75NTR, RABV tracks showed a greater processivity than that of RABV-only tracks (Fig. 7G,H), suggesting that mutual transport of RABV with NTF receptors induces a more progressive transport.

### RABV binding to p75NTR facilitates faster and more processive axonal transport

**Figure 8:**
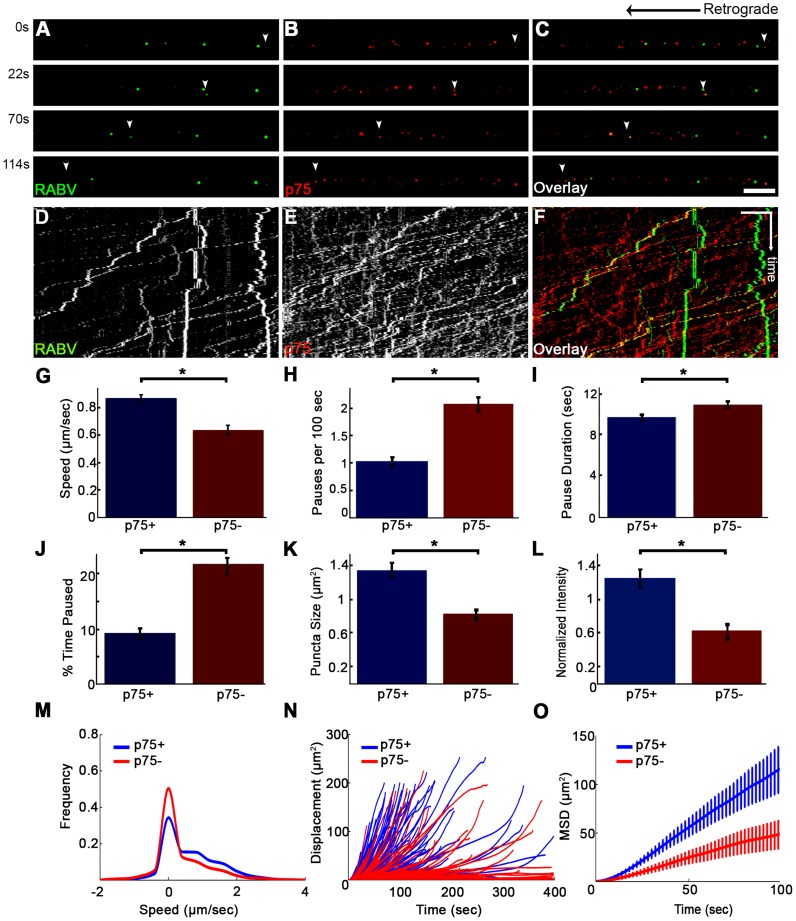
RABV travels faster and is more directed when transported with p75NTR. (**A–C**) Multi-channel live imaging of EGFP-RABV 2 hours after addition to distal axon compartment of DRG explant previously treated with a fluorescent antibody against p75NTR. Arrowheads: p75NTR-positive RABV puncta, scale bar = 10 µm. (**D,E**) Kymographs of and P75NTR extracted from time lapse depicted in (A–C). (**F**) RABV-only tracks (green) are less directed than RABV-p75NTR tracks (yellow), as shown when overlaying corresponding kymographs. Vertical scale bar = 5 µm, horizontal scale bar = 40 seconds. (**G–O**) Characterization of directed RABV puncta, transported with and without p75NTR, n = 184 and n = 122, respectively. (G) RABV presents higher speeds when transported with p75NTR, due to less frequent (H) and shorter pauses (I). Overall RABV-p75NTR spent less time paused on average (J), Diameter and intensity measurements revealed that RABV puncta positive for p75NTR were larger (**K**) and had higher intensity levels (**L**) than p75NTR-negative puncta. (**M–O**) p75NTR positive puncta (blue) are faster, more directed and present higher displacements over time, compared to p75NTR negative puncta (red), illustrated by distribution of instantaneous velocities in (M) (RABV+p75: n = 8051 events; RABV-p75: n = 7423 events) displacement plotted over time (N) and mean square displacement (O). Data is pulled from two separate experiments, error bars represent SEM. *p<0.05.

Although p75NTR may serve as a receptor for RABV (Fig. 4 and Movie S5) and the viral G-protein binds with high affinity to the p75NTR, the receptor is not an absolute requirement for RABV infection, as RABV can also infect p75NTR deficient cells [25]. Furthermore, RABV could be transported retrogradely along the axon without p75NTR (Fig. 7). To assess the precise contribution of p75NTR to RABV transport, we tracked the transport of RABV particles along the axon, with and without p75NTR (Fig. 8 A–C and Movie S8). Plotted kymographs of RABV and p75NTR demonstrate that motile RABV tracks tend to co-localize with those of p75NTR (Fig. 8D–F). Separate characterization of each group's transport (Fig. 8G–O) demonstrated that when traveling with p75NTR, RABV particles traveled faster compared to particles negative for the receptor, with respective speeds of 0.86±0.04 versus 0.63±0.04 µm/sec (Fig. 8G). We attribute this alteration in speed to the fact that RABV-p75 puncta are less prone to pausing on their route to the cell body, with an average of 0.9±0.1 vs. 2.3±0.2 pauses per 100 seconds for the p75NTR positive and negative groups, respectively. Moreover, p75 positive particles paused for shorter times (Fig. 8I) and overall spent less time paused during their travel (Fig. 8J). Another factor contributing to their higher respective speed was their instantaneous velocities. The distribution of instantaneous velocities of RABV particles positive for p75NTR is shifted towards the higher velocities when compared to RABV negative for p75NTR. RABV(+)p75NTR average velocity was higher than that of RABV(−)p75NTR, 0.71±0.008 versus 0.49±0.007 µm/sec (n = 5494 and 3919, respectively). Another interesting finding was that less than 10% of the recorded velocity events in the p75NTR positive group were anterograde, while over 17% of events in the p75NTR negative groups were anterograde, i.e. moving “backwards” towards their entrance area, the axon tip. This implies that p75NTR not only has a role in assisting the transport of NTF and viruses, but might also regulate the transport machinery, contributing to vesicle directionality towards the cell body.

Measuring both the area and average intensities of the RABV particles in each group, we found that the p75NTR positive RABV particles were larger in size, (average area of 1.34±0.09 µm^2^ vs. 0.81±0.07 µm^2^, p<0.0005), and had stronger intensity of GFP signal, when normalized to the average intensity of RABV particles in each experiment (1.24±0.11 vs. 0.61±0.1, p<0.001) (Fig. 8K–L). These larger and more prominent particles possibly represent larger endosomes, containing several RABV particles and receptors. These particles, positive for p75NTR, cover a greater net distance per time unit and are more directed towards the cell body, as shown when we compared their trajectories and mean squared displacements (Fig. 8N and O, respectively). Taken together, the major differences that were observed between the two RABV groups suggest distinguishable transport mechanisms. It therefore seems that RABV binding to p75NTR allows the virus to exploit a rapid transport mechanism to facilitate its trafficking to the cell body.

We continued to examine the role of p75 in RABV transport by tracking RABV in axons of a DRG explant after p75 knock-down. DRG explants were grown in microfluidic chambers and infected with LV-shRNA-p75-EGFP. mCherry-RABV was added to the distal channel as described before, and after 1 hour of incubation imaged for 1–2 hours. Although many axons crossed the grooves to the distal channel, only few were found to express GFP, hence were infected with sh-p75. Unlike in non-infected axons, where RABV was easily identified when trafficked towards the cell body, RABV transport in sh-p75 axons was less frequent (Fig. S4A–C). The number of transported RABV particles was reduced in sh-p75 axons when compared to adjacent, non-infected axons or to LV-EGFP infected controls (Fig. S4D) or to LV-EGFP infected controls (not shown). The few RABV particles in sh-p75 axons were less directed than those transported in non-infected cells, as seen by their respective trajectories (Fig. S4E).

## Discussion

**Figure 9:**
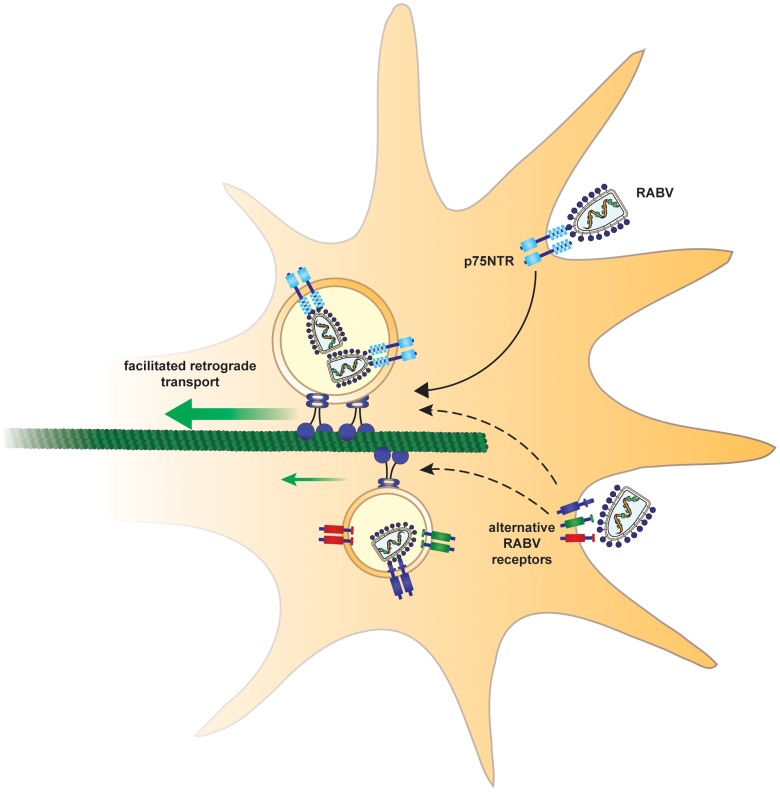
Suggested model. In order to arrive at the cell body and subsequently the CNS, rabies virus hijacks a fast route using the p75NTR endosomal pathway. In a p75NTR dependent path, RABV manipulates the axonal transport machinery to migrate faster to the cell body. An alternative, slower path, may involve alternative RABV receptors.

We have shown that fast, processive RABV retrograde transport along the axon is mediated via binding to p75NTR (Fig. 8,9). RABV is internalized at the axon tip, in part by exploiting the p75NTR pathway (Fig. 3,4). Moreover, RABV not only hijacks the neurotrophic factor endocytosis and retrograde transport machinery, but apparently its binding to p75NTR affects the axonal transport process. This interaction triggers a faster and more efficient way for RABV to reach the cell body. RABV pauses fewer times and its instantaneous velocities are significantly higher, when localized with p75NTR. Indeed, *in vivo* peripheral infection of RABV, demonstrates that the population of large DRGs, which are positive for p75NTR, is preferentially infected [29]. These findings show that p75NTR may act as an entry receptor for RABV and target internalized viral particles to the retrograde axonal transport machinery. Moreover, we show that receptor utilization in neurons can determine the velocity of retrograde axonal transport. These findings reveal how RABV can spread from the periphery, describe a novel role for the RABV-p75NTR interaction beyond ligand-receptor binding, and define a mechanism that allows proficient long-distance accelerated transport in axons.

### Recruitment of RABV to the retrograde transport machinery

A crucial initiating event for the mechanism outlined above is the binding of RABV to p75NTR. Here we provide direct evidence that p75NTR may serve as a receptor for internalization at axons tips, as well as mediate incorporation into the endosomal neurotrophic transport pathway. However, RABV does not strictly rely on p75NTR for internalization and may enter the cell in a p75NTR independent pathway, while is also known to bind other receptors [20], [30]. Hence there are likely to be additional ways for RABV to merge into the p75NTR-RABV endosome. Indeed, we observed events where RABV particles merge with p75NTR-positive endosomes en route (Movie S9). The p75NTR neurotrophin receptor accelerates RABV transport to the cell body, yet there are instances of fast, processive transport of RABV particles without the receptor. We assume other identified RABV receptors such as NCAM [30] or other, un-identified ones, may facilitate RABV's retrograde axonal transport within endosomes in a similar fashion. Our experiment with DRG cultures where p75 was knocked down, show reduced RABV infection and transport (Fig. 4 and Fig. S4), and further support the role for p75NTR proposed here.

Some viruses such as Herpes Simplex Virus can travel along the axon independently of a membrane compartment, as capsids [31] and control its long distance transport process directly [32]–[35]. Interestingly, the RABV phosphoprotein P directly interacts with a dynein light chain [11], [12], suggesting a mechanism whereby this interaction is key to RABV's retrograde trafficking. However, studies on the retrograde transport of RABV enveloped virions [14] and infection of the CNS from the periphery with dynein light chain binding defective virus mutants [13] already showed that such an interaction is not essential for retrograde axonal transport of the virus. Our data support this finding, as we demonstrated that RABV is transported in acidic compartments (Fig. 6), and mostly in p75NTR-positive endosomes (Fig. 7). A different role for dynein binding should thus be considered. Dynein is well characterized as a retrograde motor, yet can also act to tether and stabilize dynamic microtubules [36]. Possibly, RABV binding to dynein tethers projecting microtubules (MT) in the cell cortex thereby facilitating its retrograde trafficking from the cell periphery. Following this tethering, RABV particles can merge into the RABV-p75NTR endosomes and travel to the neuron cell body. Nonetheless, this RABV-MT interaction could be mediated by binding of RABV to NCAM, which was demonstrated to tether MT's at the synapse [37]. Suggestions relating to the function of various RABV populations that can either internalize with receptors to endosomes, or act as RNP capsids to manipulate and stabilize the cytoskeleton, require further testing.

### RABV is transported in an acidic compartment

We have shown that the RABV and the acidic marker LysoTracker are co-localized and move together along the axon. This shows that the RABV is transported in membranal compartments similar to neurotrophic factors and is unlikely to be free in the cytoplasm. Interestingly, low pH induces conformational changes in the viral G protein, suggesting control of membrane fusion events [38], [39] that may regulate RABV transport in acidic vesicles. Moreover, it could be that the alteration in RABV G-protein function, as pH changes, provides a signal for RABV-p75NTR complex, leading to its transport acceleration.

### Potential regulatory roles for the RABV-p75NTR interaction

The effects of RABV binding to p75NTR on axonal transport processivity and speed, suggest that down-stream signaling is activated and exerts an influence on the retrograde transport process. Axonal transport can be regulated at four main different levels: 1. Microtubule tracks. 2. Motor proteins. 3. Motor-cargo adaptors. 4. ATP supply. As we have shown that RABV-p75NTR complexes move both instantaneously faster and with fewer pauses, we speculate that more than one regulatory level may be involved. p75NTR activates different signaling cascades, as a result of binding to several distinct ligands or interactions with various co-receptors. Recently it was described that structural determinants underlie the signaling specificity of p75NTR to the JNK, RhoA and NF-kB pathways [40]. Interestingly, c-Jun N-terminal kinases (JNKs) can also regulate the axonal transport process in several different ways. JNK-interacting proteins (JIPs) are scaffolding proteins for JNK and serve as linkers between motor proteins and their membrane-associated cargos. JIP1 serves as a linker between kinesin-1 and dynein to vesicles, and JNK signaling can modulate its transport by regulating the two opposing motors [41]. JNK, by functioning as a kinesin-cargo dissociation factor, regulates axonal transport [42]. Additionally, JNK3 phosphorylates kinesin-1 and inhibits its microtubule-binding activity [43], [44]. JNK3 and its scaffolding protein Sunday driver (syd) are activated after axonal injury and bind to p150, the regulatory sub-unit of the dynein-dynactin retrograde complex [45]. Moreover, a recent study has shown that JIP1 phosphorylation serves as a molecular switch to regulate the direction of vesicle transport in neurons, by coordinating kinesin and dynein motors [46]. These studies show that scaffolding proteins such as the JIPs and JNK play an important role in the regulation of motor proteins and the axonal transport process, and thus might take part in RABV manipulation of the axonal transport machinery.

Another interesting speculation to explain how RABV binding to p75NTR accelerates its transport is the potential involvement of axonal activated NF-kB. NF-kB can be activated downstream to both p75NTR and RABV in the axon before entering the nucleous, and may affect dynein activity [47]–[49]. Interestingly, NF-kB activation after NGF binding to p75NTR enhances neuronal survival [50], suggesting that p75NTR may regulate NGF retrograde signaling. In the future, it will be interesting to examine the involvement of axonal p75NTR dependent downstream signaling activated by RABV and its effect on the neuronal cytoskeleton as well as axonal transport.

It is also tempting to consider that local protein synthesis as the result of RABV binding to p75NTR can facilitate this processive transport. Indeed recently it was demonstrated that efficient retrograde transport of pseudorabies virus, require axonal protein synthesis [51].

These data reveal an unexpected role for p75NTR that is not necessarily related to its functions as a neurotrophin receptor, but rather to acceleration of RABV-axonal transport. Whether p75NTR might also provide a fast delivery of other axonal cargos, such as neurotrophic factors, pathogenic prions and tetanus toxin [1] is a question for future research.

### Long-distance axonal transport of cargo

This study has addressed the question of how RABV is transported over long distances. Previous cell biology work in the field was performed mostly on RABV-infected cell lines or neuronal cell bodies, leading to a focus on mechanisms of infection and not on long distance transport [52]. Although neurotropic viruses need to progress over long distances to reach the CNS [1], [35], how RABV performs this task was not clear. Here, we suggest that RABV hijacks a specific mechanism that enables the neuron to transport cargos over long distances. Interestingly, p75NTR is internalized by clathrin dependent endocytosis and is sorted into distally transported endosomes after stimulation with NGF [53], [54]. Furthermore, RABV internalization was characterized as a dominantly clathrin mediated process [52], [55]. Here we show that RABV binds to and is internalized together with p75NTR, forming endocytic compartments which undergo processive long distance transport. This suggests similar mechanism by which RABV mimics neurotrophins for activation of p75NTR ligand-mediated internalization and transport. As p75NTR can bind many ligands and various co-receptors, it is possible that binding to some will trigger a signaling effect that will facilitate axonal transport of other cargos and not only RABV. As we demonstrate here for RABV, p75NTR interaction can modulate the cellular transport machinery and may serve as a novel route to increase transport efficiency and facilitate arrival of cargos to the CNS from the periphery.

## Materials and Methods

### Animals and Dorsal Root Ganglion explants

ICR mice were bred and maintained at the Tel Aviv University animal care facility until the time of sacrifice. Spinal cords were dissected from E12.5–13.5 mice, followed by separation of Dorsal Root Ganglia (DRG) from meninges and additional spinal cord. The Institutional Animal Care Committee at the Tel Aviv University approved all the animal protocols in this work.

### Microfluidic chamber preparation

Microfluidic chambers were fabricated using methods previously described in detail [56]. All microfluidic chambers were replica molded using PDMS (#41201841 Dow Corning) from masters that were patterned using the photosensitive epoxy SU-8 (Microchem). All masters consisted of two permanent SU-8 layers on a 3″ silicon wafer and were made in the clean room facility in Tel-Aviv University. The first layer of SU-8 (3 µm depth) contained the microgrooves, which were patterned by photolithography using a high-resolution chromium mask (5 µm minimum feature size; Advance Reproduction Corp.). The second layer of SU-8 (100 µm depth) contained the compartments, which were patterned by photolithography using a 20,000 dpi printed transparency mask (CAD/Art Services, Inc.). Chamber dimensions: channels: length 8.25 mm, width 1.5 mm; grooves: length 400 µm, width 15 µm, height 5 µm (Fig. 1B). A single 7 mm well was punctured into the “proximal” or explant channel, into which a “cave” was carved using a scalpel, to prevent explants from floating, 2 additional 1.2 mm wells were punctured into the channel on either side of the “explant” well to allow flow. Two 7 mm wells were punctured into both ends of the “distal” or axons channel to allow control over the distal channel. Microfluidic devices were cleaned of surface particles using adhesive tape and sterilized in 70% high-grade ethanol for 1 h. Devices were allowed to completely air dry under sterile conditions, attached to sterile 50 mm glass bottom dishes (FD5040-100, WPI) using gentle pressure and heated to 70°C for 20′ to improve adhesion to glass. Chambers were coated using 150 µl of 1.5 ng/ml Polyornithine (P-8638, Sigma) in PBS for 24 hours, which was replaced with 150 µl Laminin (L-2020, Sigma) 1∶333 in DDW for 24 hours. Laminin was replaced with culture medium until plating (1–3 days).

### Assembly and maintenance of DRG explants

At the day of plating, media were removed from all wells, and a single DRG was inserted to each explant “cave” using a 20 µl tip. Following 1 hour of incubation at 37°C, 150 µl of medium were added to each well. Basic culture medium consisted of Neurobasal medium (Life Technologies) supplemented with 2% B-27 (Life Technologies), 1% penicillin-streptomycin (Biological Industries, Israel) and 1% Glutamax (Life Technologies). A gradient of murine NGF was created in order to encourage axons to cross the grooves to the distal axon compartment, by adding 100 ng/ml and 62.5 ng/ml murine NGF (Alomone labs) to the distal and proximal wells, respectively. Cultures were maintained at 37°C and 5% CO_2_, and media were refreshed every 2 days. Transport assays were initiated after axons had crossed the grooves and established an axonal network in the distal compartment, 3–5 days from plating (Fig. 1D). For TIRF imaging assays, 4–5 DRG were placed on 35 mm glass bottom dishes (FD35-100, WPI) with 20 µl each of DRG culture medium supplemented with 62.5 ng/ml NGF. DRG were incubated in 37°C for 4–5 hours to allow adhesion to glass, then supplemented with 2 ml DRG culture medium supplemented with 62.5 ng/ml NGF. Cultures were maintained at 37°c and 5% CO_2_ for 48 hours before imaging.

### Fluorescent EGFP/mCherry rabies virus production

rRABV EGFP-P ΔG/rRABV mCherry-P ΔG are recombinant rabies viruses in which EGFP or mCherry fluorescent reporters were fused to the phosphoprotein P and in which the glycoprotein G gene was deleted. Preparation and amplification was performed as previously described [14], [57]. In brief, MG-136 cells, expressing the RABV matrix and glycoproteins after induction with doxycycline, were infected with rRABV EGFP-P ΔG. Cells were split, after which media were twice replaced with fresh medium supplemented with 1 mg/ml Doxycycline, to be collected 48 hours later. rRABV EGFP-P ΔG was concentrated from cell culture supernatants using the PEG virus precipitation kit (ab102538, Abcam).

### EGFP/sh-RNA-p75-EGFP lentivirus production

HEK293t cells were transfected using calcium-phosphate precipitation with the viral vector pLL-EGFP and the helper plasmids pVSVG and pGag-PolGpt (kind gift from Eran Bacharach) or mix of 4 shRNA plasmids against murine p75, produced from Mouse GIPZ lentiviral target gene shRNAmir glycerol set (GE healthcare RMM4532-EG18053). Viral particles were collected 48 and 72 hours after transfection, filtered and concentrated using the PEG virus precipitation kit (ab102538, Abcam).

### DRG cultures, shRNA-p75 and RABV infection

DRG were collected from E12.5–13.5 mice, trypsinized with Trypsin-EDTA solution B (Biological industries 03-052-1B) for 5 minutes in 37°C, and washed with complete F12 medium. Cells were dissociated by pipetting, counted and plated on PLO/Laminin coated 96 well plates, in a density of 15K cells per well. Cells were maintained with DRG culture medium supplemented with 62.5 ng/ml NGF. On day of plating, cells were infected with either LV-EGFP or a mix of 4 LV-shRNA-p75-EGFP (MOI≈5 and MOI≈10, respectively, due to high infectivity of LV-EGFP). 3–4 days from plating, cells were infected with ≈120K mCherry RABV particles for 0′, 30′ and 120′ in duplicates after which they were washed ×3 with DRG culture medium. 48 hours later, neurons showing obvious mCherry foci were counted in 6–9 fields per well, and divided by total number of counted neurons. Although cultures varied in axon network, non-neuronal cell populations and LV-infection levels, infection rates were lower in sh-RNA-p75-EGFP groups when compared to GFP control in all experiments. Due to differences in absolute infection rates, these were normalized to the rate obtained at the LV-EGFP control group.

Infection of DRG explants in microfluidic chambers were performed on the day of plating, with ≈1×10^6^ LV-EGFP particles, or a mix of 4×≈1–2×10^6^ LV-sh-RNA-p75-EGFP particles.

To estimate knockdown of p75, a DRG culture plated on a glass bottom dish was infected with sh-RNA-p75-EGFP as described above. At 4DIV, culture was treated with 1∶100 anti-p75-550 for 15′ and washed ×3 prior to imaging using spinning disc confocal.

### Fluorescent beads, markers and antibodies

Qdot-NGF was prepared by mixing biotinylated murine-NGF 10 µg/ml (#N-240-B, Alomone Labs) with Quantum-Dot 605 streptavidin conjugate 1 µM (Q10101, Molecular Probes) in a molar ratio of 3∶1, respectively.

Acidic compartments were delineated using Lysotracker Red DND-99 (Life Technologies) in a final concentration of 50 nM. Mitotracker Deep Red FM (Life Technologies) was used to denote mitochondria, in a final concentration of 100 nM.

In order to track the co-transport of RABV with its receptors, the fluorescent extracellular antibodies Anti-p75NTR-ATTO-550, Anti-p75-ATTO-488 and Anti-TrkA-ATTO-633 (ANT-007-AO, ANT-007-AG and ANT-0180-FR, respectively, Alomone Labs) were used in a 1∶100 dilution.

### Live imaging of axonal transport

Live imaging was performed on DRG explants grown in compartmental chambers, 3–5 DIV, upon forming an axonal network at the distal axon compartment. Prior to imaging, media from each well were replaced with 150 µl Neurobasal medium supplemented with 1% penicillin-streptomycin and 1% Glutamax (poor medium). Following two hours of starvation, 30 µl of poor medium were added to the proximal well, to induce compartmental separation. 2 µl of concentrated EGFP-RABV (≈120K particles), Qdot-NGF or both were added to one distal well, with additional 5 µl medium, to encourage flow through the distal axon channel (Fig. 1B,C). Chambers were incubated for 1–2 hours in 37°C and 5% CO_2_ prior their placement on the microscope stage.

Time lapse images of axons in the groove area (Fig. 1B,C), were acquired at 37°C and CO_2_ controlled environment, using Nikon Eclipse Ti microscope equipped with Yokogawa CSU X-1 spinning disc confocal, controlled via iQ software (Andor). Multi-channel time lapses were captured using 60× lens, NA = 1.4 with 2000 msec intervals, with an approximate lag of 100–400 ms between channels. Digital images were taken with Andor iXon DU-897 EM-CCD camera.

### Axonal transport image analysis

Time-lapse image analysis was carried out using Fiji, following subtraction of average intensity z-projection to exclude completely stationary fluorescent artifacts. XY coordinates of tracks were registered from 9 or more grooves (which contains typically 2–5 axons) in each experiment, using Manual Tracking plugin, while distances, velocities and MSD's were computed using MATLAB implementation for Fiji Trackmate plugin [58]. Tracks with run lengths <10 µm and/or average speed <0.2 µm/sec were filtered out of the analysis. A single puncta traveling with instantaneous velocity <0.1 µm/sec for three or more consecutive frames (i.e 6 seconds), was considered paused. Instantaneous velocities were calculated as the distance a puncta traveled between two consecutive frames, divided by frame interval (2 sec). Speed distribution was presented for 0.2 µm/sec bins, and data were interpolated to include 10^3^ points. Track speed was calculated as total run length divided by track duration, while displacement considered the net-distance a puncta traveled in retrograde direction. Kymographs were drawn using the Kymotoolbox plugin [59]

In order to quantify co-localization of RABV with receptors and cellular components, RABV-EGFP images threshold was manually set, then RABV puncta detected using Fiji particle analysis feature, and saved as ROI's. ROI's were measured using Fiji measure function to illustrate both puncta diameter (area) and GFP intensity. Intensities were normalized to the average intensity of RABV puncta in each experiment. Images were subsequently matched with corresponding images from requested channel, and occurrences of co-localized puncta inside ROI's were manually counted. For random co-localization, RABV ROI's were matched with a random image from the same channel and time lapse.

### TIRF imaging of RABV/NGF internalization

DRG explants were imaged with TILL Photonics iMIC TIRF microscope (FEI, Munich, Germany), using Olympus 100× NA 1.49 TIRF objective. Images were acquired with an Andor iXon 897 EMCCD camera (Belfast, UK) and imaging protocol was controlled by TILL Photonics LiveAcquisition software. Throughout the experiment, explants were imaged inside an environmental chamber maintaining 37°C and 5% CO_2_. TIRF angle was set to give minimal penetration of the evanescent wave still giving measurable signal from the RABV-EGFP/NGF-QD particles. We used the 360° TIRF feature which azimuthally spins the laser beam on the circumference of the objective back focal plane, creating homogenous TIRF illumination across the field. Broad neuronal tips were selected for imaging after RABV/NGF addition, selected fields were imaged for 5–10 minutes each, for up to 2 hours. Exposure times were 50 msec, and camera gain was set to 300. Final frame rate was 1 and 1.12 seconds per frame in RABV/NGF alone and RABV+p75NTR experiments, respectively. A frame is comprised of RABV/NGF/p75 fluorescent channels over a bright-field image to localize fluorescent particles in context. For RABV-p75 imaging, explant cultures were incubated with fluorescent anti-p75NTR (ANT-007-AO, Alomone Labs) for 10 minutes and washed 3 times in poor neurobasal medium prior to imaging.

### Measurement and analysis of RABV/NGF and p75 internalization in TIRF

Time-lapse image analysis was carried out using Fiji. RABV/NGF/p75 particles, which were manually identified in the area of the neuronal tip according to bright-field images, were marked by circular ROI in every time point. Average intensity was measured and plotted over time. A three-point moving average was applied to smooth out noisy spiking. As movement of RABV/NGF particle farther from the evanescent wave was measured as reduction in signal intensity, we used this property to detect and illustrate internalization kinetics. More specifically, internalization was defined as gradual reduction of RABV/NGF signal to undetectable levels for more than 4 consecutive frames (4–4.5 seconds). Internalization time was calculated as the time of the gradual reduction from high level intensity to baseline level. In RABV-p75 co-labeling experiments, p75 intensity was measured inside the identified RABV ROI.

## Supporting Information

Figure S1 **No correlation found between number of particles tracked per axon and transport measurements.** Pearson's correlation tests were applied in order to determine whether correlation exists between the number of RABV puncta tracked per axon and measured transport parameters. (**A**) No strong correlation was found between the number of tracks and the rate of directed-ness (directed: run lengths >10 µm and average speed >0.2 µm/sec) (**B**), average track speed (**C**), track displacement or (**D**) run length.
(TIF)

Figure S2 **Close proximity of p75 and RABV at the tip of DRG axon visualized by STED microscopy.** DRG explants cultured on coverslips were infected at 2DIV with either EGFP or mCherry-labeled RABV (≈120K viral particles) for 1 hour, stained with either fluorescent rabbit anti-p75-550 antibody or unlabeled antibody followed by fluorescent anti-rabbit conjugated with Alexa-488. DRG explants were then fixed with 4% PFA, mounted and imaged with a Leica-TCS-STED confocal equipped with 592 nm CW laser in STED mode, enabling super resolution imaging of fluorophores emitting at the EGFP spectrum. (**A**) p75NTR is labeled with fluorescent antibody imaged in normal confocal mode, RABV-EGFP is imaged in STED mode. (**B**) 2×2 µm zoom-in images of the RABV-p75 co-localized spots shown in dashed square. (**C**) RABV-mCherry is imaged in regular confocal mode, p75NTR is antibody labeled by Alexa-488 and is imaged in STED mode. (**D**) 2×2 µm zoom-in images of the RABV-p75 co-localized spots shown in dashed square. Scale bars = 5 µm.
(TIF)

Figure S3 **p75NTR knockdown in DRG culture.** (**A–C**) Dissociated DRG cultures infected with LV-sh-RNA-p75-EGFP, were treated with anti-p75-550 for 15′, and then washed 3 times. sh-RNA positive axons (arrowheads) did not present staining with p75 antibody as seen in-non infected axons (arrows).
(TIF)

Figure S4 **p75NTR knockdown reduces transport of RABV.** DRG explant grown in microfluidic chamber was infected with LV-sh-p75-EGFP. mCherry-RABV was applied to the distal channel at 5 DIV for 2 hours. (**A–C**) Dual color live imaging reveals that fewer particles were transported in sh-p75 axons (full arrowheads) as opposed to adjacent non-infected axons (outlined arrowheads). (**D**) More RABV puncta were transported in non-infected or LV-EGFP axons than in sh-RNA-p75 axons, over a period of 400 seconds (n = 4 axons each). (**E**) Trajectories of RABV particles from non-infected axons (blue, n = 9 particles) show greater processivity and displacement than those of RABV particles in sh-RNA-p75 axons, in the same culture (red, n = 5 particles).
(TIF)

Movie S1 **EGFP-RABV retrograde axonal transport.** Fluorescent viral particles applied to distal DRG axons cultured in a compartmentalized chamber. Live imaging was performed with spinning disc confocal using a 60× lens at a rate of 0.5 frames per second. Playback speed is 10 frames per second.
(AVI)

Movie S2 **NGF retrograde axonal transport.** Quantum-dot-labeled NGF applied to distal DRG axons culture in a compartmentalized chamber, live imaged with spinning disc confocal, 60× lens at a rate of 0.5 frames per second. Playback speed is 10 frames per second.
(AVI)

Movie S3 **EGFP-RABV internalization at axon tips.** Fluorescent viral particles are internalized (arrow) at the growth cone of a DRG explant. Live TIRF imaging using 100× lens at a rate of 1 frame per second. Playback speed is 10 frames per second.
(AVI)

Movie S4 **NGF internalization at axon tips.** Quantum-dot-labeled NGF is internalized (arrow) at the growth cone of a DRG explant. Live TIRF imaging using 100× lens at a rate of 1 frame per second. Playback speed is 10 frames per second.
(AVI)

Movie S5 **EGFP-RABV and p75NTR are internalized together at axon tips.** Fluorescent viral particles (red) and fluorescent antibody-labeled p75NTR (green) are co-localized while internalized into the DRG axonal growth cone (arrows). Live TIRF imaging using 100× lens at a rate of 1 frame per second. Playback speed is 10 frames per second.
(AVI)

Movie S6 **EGFP-RABV and NGF are co-transported in axons.** Fluorescent viral particles (green) and Quantum-dot-labeled NGF (magenta) applied to distal DRG axons cultured in a compartmentalized chamber are transported together (white) towards the cell body. Live imaged using spinning disc confocal with a 60× lens at a rate of 0.5 frames per second. Playback speed is 10 frames per second.
(AVI)

Movie S7 **EGFP-RABV is transported with neurotrophin receptors in axons.** Fluorescent viral particles (green) applied to distal DRG axons grown in a compartmentalized chamber, along with fluorescent antibody-labeled p75NTR (red) and TrkA (blue). RABV and receptors are mutually transported towards the cell body, often in the same compartment. Live imaged using spinning disc confocal with a 60× lens at a rate of 0.5 frames per second. Playback speed is 10 frames per second.
(AVI)

Movie S8 **EGFP-RABV is transported faster when linked to p75NTR.** Fluorescent viral particles (green) applied to distal DRG axons grown in a compartmentalized chamber, along with fluorescent antibody-labeled p75NTR (red). RABV is mostly transported with p75NTR (filled arrows), however non-p75 dependent transport is also visible (outlined arrow). Live imaged using spinning disc confocal with a 60× lens at a rate of 0.5 frames per second. Playback speed is 10 frames per second.
(AVI)

Movie S9 **EGFP-RABV can encounter p75NTR en route to the cell body.** Fluorescent viral particles (green) applied to distal DRG axons grown in a compartmentalized chamber, along with fluorescent antibody-labeled p75NTR (red). A single RABV particle (arrow) is stationary until arrival of p75NTR and pauses again after p75NTR leaves. Live imaged using spinning disc confocal with a 60× lens at a rate of 0.5 frames per second. Playback speed is 10 frames per second.
(AVI)
