# Supplementary figures and images for: Rabies Virus Hijacks and Accelerates the p75NTR Retrograde Axonal Transport Machinery
Source: PLoS Pathog. 2014 Aug 28;10(8):e1004348. doi: 10.1371/journal.ppat.1004348 (PMC4148448; doi:10.1371/journal.ppat.1004348)

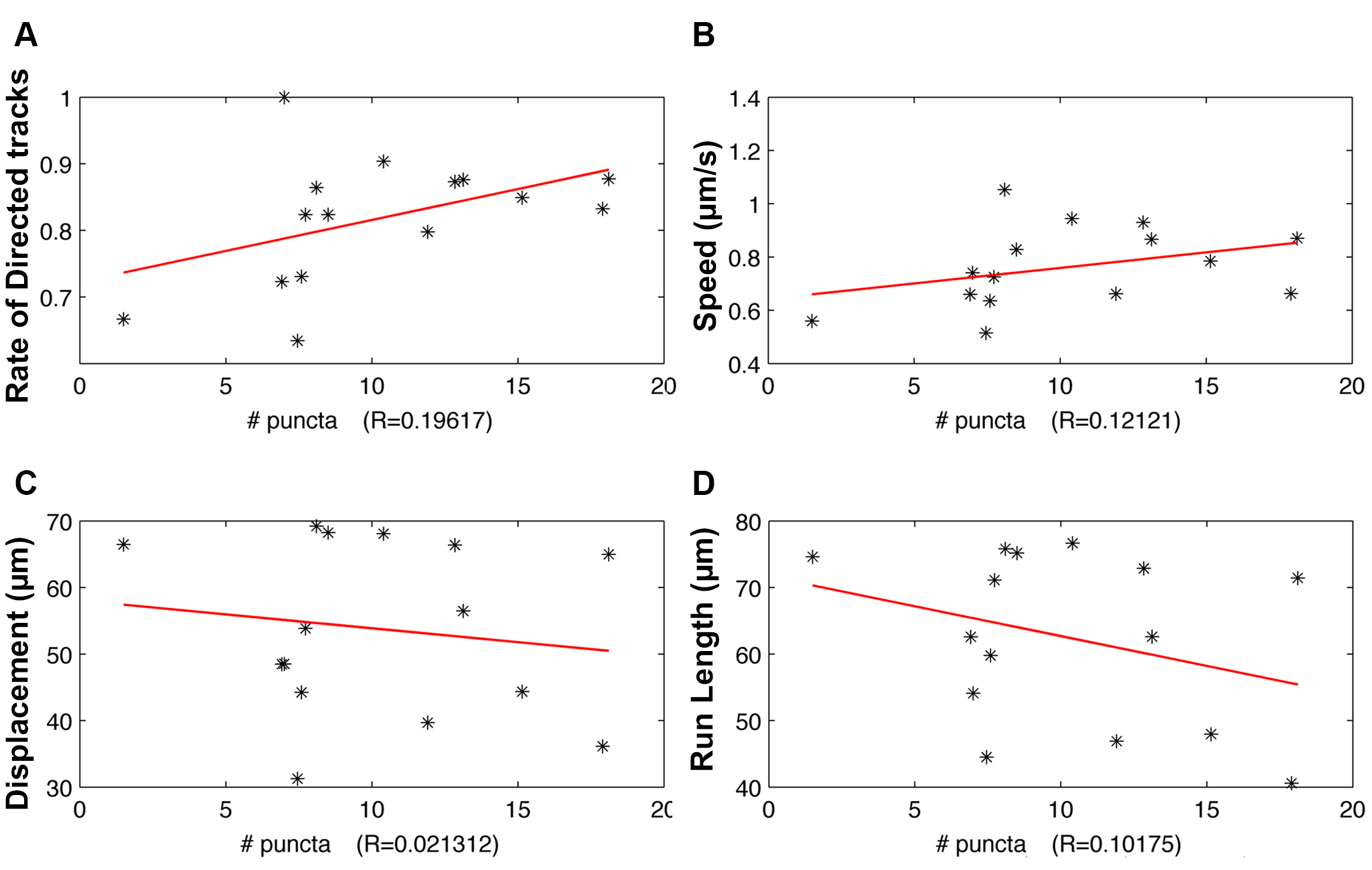

Supplement: Figure S1 — No correlation found between number of particles tracked per axon and transport measurements. Pearson's correlation tests were applied in order to determine whether correlation exists between the number of RABV puncta tracked per axon and measured transport parameters. (A) No strong correlation was found between the number of tracks and the rate of directed-ness (directed: run lengths >10 µm and average speed >0.2 µm/sec) (B), average track speed (C), track displacement or (D) run length. (TIF) [file ppat.1004348.s001.tif]

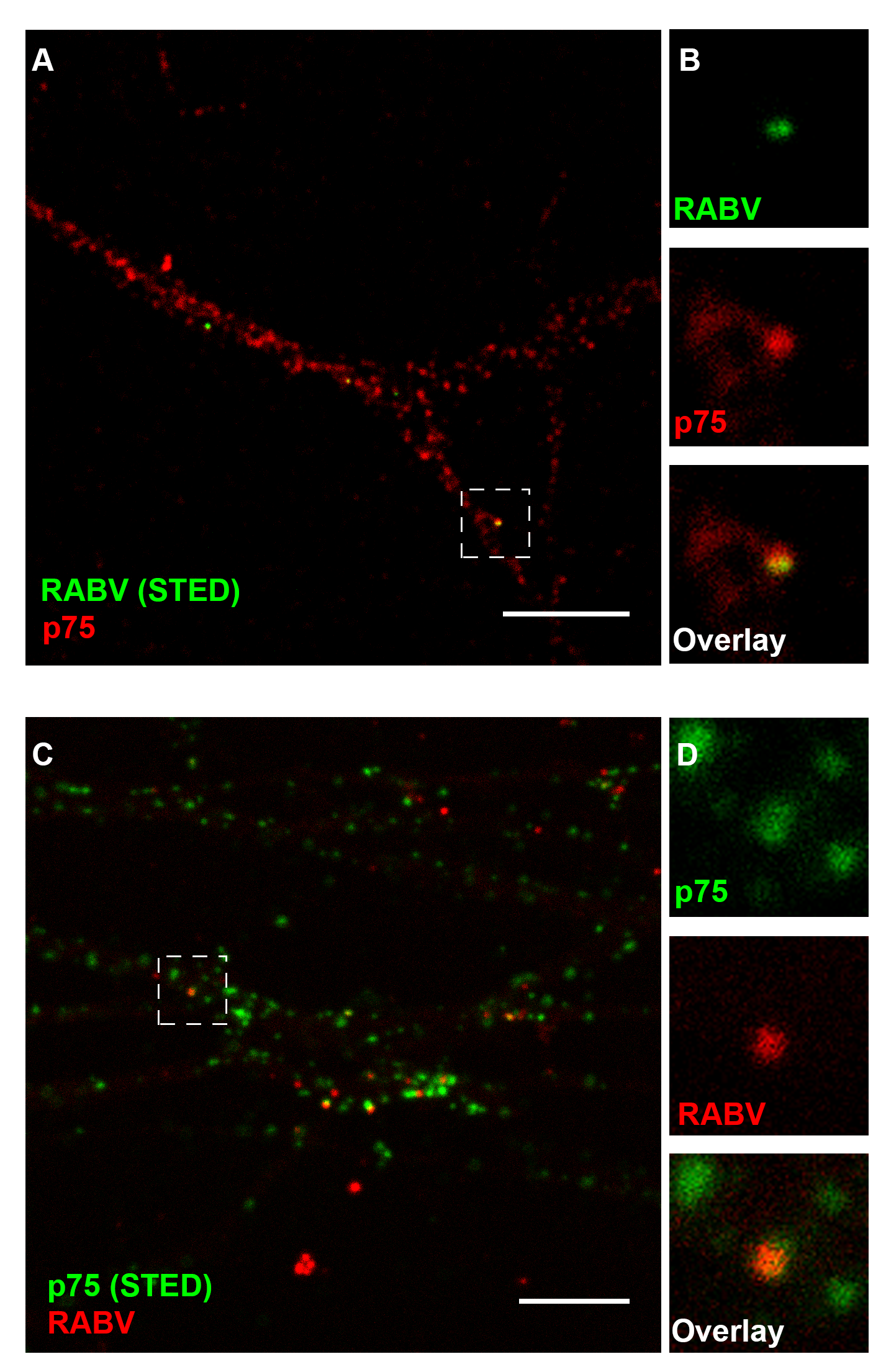

Supplement: Figure S2 — Close proximity of p75 and RABV at the tip of DRG axon visualized by STED microscopy. DRG explants cultured on coverslips were infected at 2DIV with either EGFP or mCherry-labeled RABV (≈120K viral particles) for 1 hour, stained with either fluorescent rabbit anti-p75-550 antibody or unlabeled antibody followed by fluorescent anti-rabbit conjugated with Alexa-488. DRG explants were then fixed with 4% PFA, mounted and imaged with a Leica-TCS-STED confocal equipped with 592 nm CW laser in STED mode, enabling super resolution imaging of fluorophores emitting at the EGFP spectrum. (A) p75NTR is labeled with fluorescent antibody imaged in normal confocal mode, RABV-EGFP is imaged in STED mode. (B) 2×2 µm zoom-in images of the RABV-p75 co-localized spots shown in dashed square. (C) RABV-mCherry is imaged in regular confocal mode, p75NTR is antibody labeled by Alexa-488 and is imaged in STED mode. (D) 2×2 µm zoom-in images of the RABV-p75 co-localized spots shown in dashed square. Scale bars = 5 µm. (TIF) [file ppat.1004348.s002.tif]

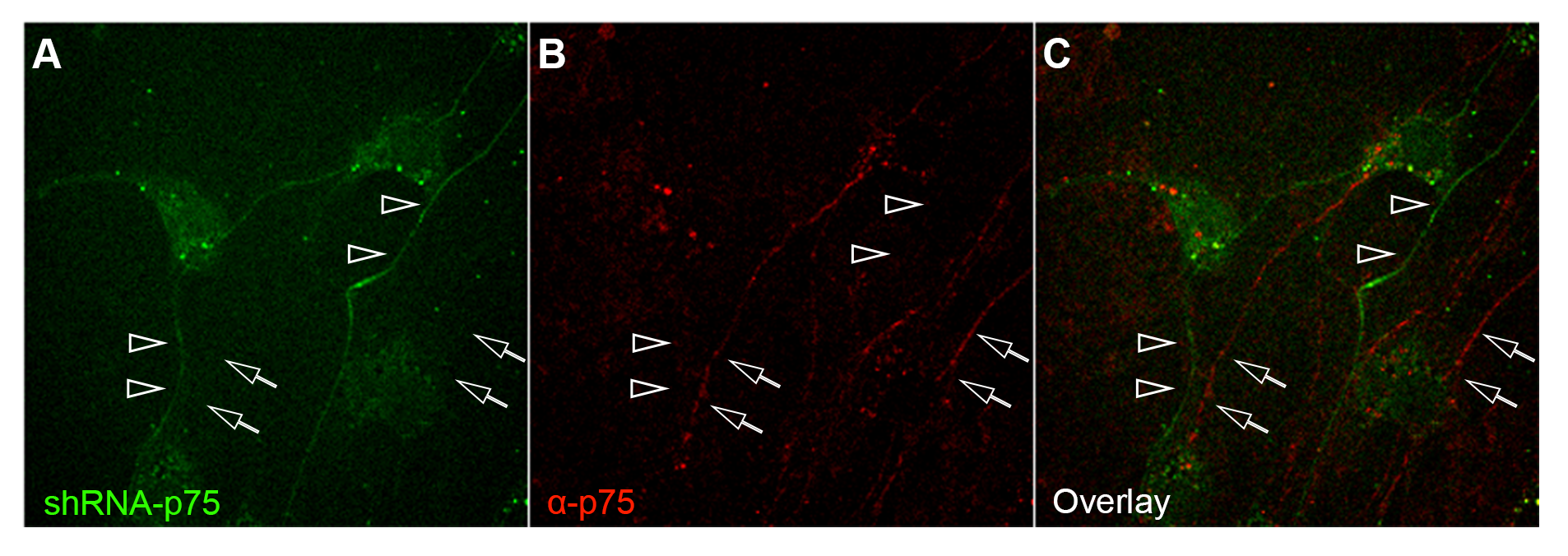

Supplement: Figure S3 — p75NTR knockdown in DRG culture. (A–C) Dissociated DRG cultures infected with LV-sh-RNA-p75-EGFP, were treated with anti-p75-550 for 15′, and then washed 3 times. sh-RNA positive axons (arrowheads) did not present staining with p75 antibody as seen in-non infected axons (arrows). (TIF) [file ppat.1004348.s003.tif]

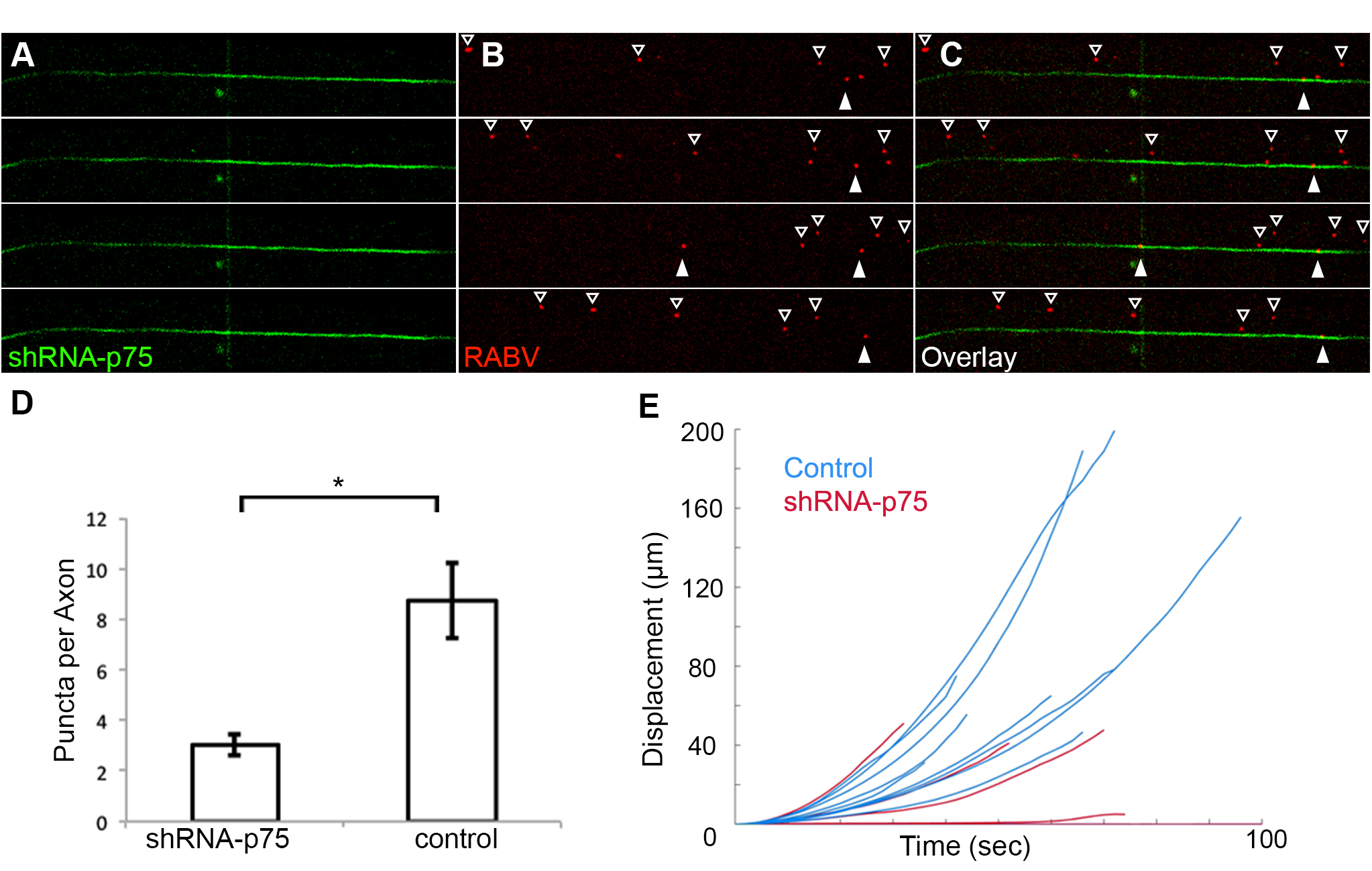

Supplement: Figure S4 — p75NTR knockdown reduces transport of RABV. DRG explant grown in microfluidic chamber was infected with LV-sh-p75-EGFP. mCherry-RABV was applied to the distal channel at 5 DIV for 2 hours. (A–C) Dual color live imaging reveals that fewer particles were transported in sh-p75 axons (full arrowheads) as opposed to adjacent non-infected axons (outlined arrowheads). (D) More RABV puncta were transported in non-infected or LV-EGFP axons than in sh-RNA-p75 axons, over a period of 400 seconds (n = 4 axons each). (E) Trajectories of RABV particles from non-infected axons (blue, n = 9 particles) show greater processivity and displacement than those of RABV particles in sh-RNA-p75 axons, in the same culture (red, n = 5 particles). (TIF) [file ppat.1004348.s004.tif]
